# Reliability and validity of the Gastrointestinal Symptom Rating Scale (GSRS) and Quality of Life in Reflux and Dyspepsia (QOLRAD) questionnaire in dyspepsia: A six-country study

**DOI:** 10.1186/1477-7525-6-12

**Published:** 2008-01-31

**Authors:** Károly R Kulich, Ahmed Madisch, Franco Pacini, Jose M Piqué, Jaroslaw Regula, Christo J Van Rensburg, László Újszászy, Jonas Carlsson, Katarina Halling, Ingela K Wiklund

**Affiliations:** 1 AstraZeneca R&D, Medical Science, Mölndal, S-431 86, Sweden; 2 Medical Department I, Technical University Hospital, Dresden, 01307, Germany; 3 Azienda Ospedaliera Careggi, U. O. di Gastroenterologia ed Endoscopia digestiva, Villa Medicea, Viale Pieraccini, 17, 50139, Firenze, Italy; 4 Servicio de Gastroenterología, Hospital Clinic de Barcelona, Villarroel 170, 08036, Spain; 5 Klinika Gastroenterologii CMKP, Centrum Onkologii, Roentgen Street 5, 02-781, Warszawa, Poland; 6 Gastroenterology Unit, Tygerberg Hospital, Cape Town, 7505, South Africa; 7 Semmelweis Hospital, Internal Medicine, Csabai Kapu 9-11, 3501, Miskolc, Hungary

## Abstract

**Background:**

Symptoms of dyspepsia significantly disrupt patients' lives and reliable methods of assessing symptom status are important for patient management. The aim of the current study was to document the psychometric characteristics of the Gastrointestinal Symptom Rating Scale (GSRS) and the Quality of Life in Reflux and Dyspepsia questionnaire (QOLRAD) in Afrikaans, German, Hungarian, Italian, Polish and Spanish patients with dyspepsia.

**Methods:**

853 patients with symptoms of dyspepsia completed the GSRS, the QOLRAD, the 36-item Short-Form Health Survey (SF-36) and the Hospital Anxiety and Depression scale.

**Results:**

The internal consistency reliability of the GSRS was 0.43–0.87 and of the QOLRAD 0.79–0.95. Test-retest reliability of the GSRS was 0.36–0.75 and of the QOLRAD 0.41–0.82. GSRS Abdominal pain domain correlated significantly with all QOLRAD domains in most language versions, and with SF-36 Bodily pain in all versions. QOLRAD domains correlated significantly with the majority of SF-36 domains in most versions. Both questionnaires were able to differentiate between patients whose health status differed according to symptom frequency and severity.

**Conclusion:**

The psychometric characteristics of the different language versions of the GSRS and QOLRAD were found to be good, with acceptable reliability and validity. The GSRS and QOLRAD were found to be useful for evaluating dyspeptic symptoms and their impact on patients' daily lives in multinational clinical trials.

## Background

The definition and characterization of dyspepsia continue to challenge clinical investigators. At the Rome II consensus conference held in 1999, the recommended definition of dyspepsia was 'pain or discomfort centred in the upper abdomen' [1,2]. Yet, symptoms such as heartburn, nausea, post-prandial fullness, early satiety or bloating may also be present [3-7]. Recent guidelines suggest that gastroesophageal reflux disease (GER) is the likely diagnosis if heartburn is the only presenting symptom or the predominant symptom [8-10].

The label 'functional dyspepsia' is often used interchangeably with that of 'dyspepsia', but actually refers to patients whose dyspepsia has been investigated and has no known structural or biochemical cause [8,11]. Studies on the prevalence of dyspepsia in primary care originate mainly from a few developed countries, where the disorder accounts for 1–4% of all primary care consultations [6,12,13]. Estimates of the prevalence of dyspepsia in the adult general population are noticeably higher, ranging from 20–40% [7,11]. The frequency of medical visits for dyspepsia has been found to increase with increasing age [14], and with increasing severity of dyspeptic symptoms [15].

Dyspepsia symptoms impair patients' daily functioning [16-21]. The symptom-driven nature of patient management places particular importance on a reliable estimation of symptom status. Hence, the assessment of how symptoms of dyspepsia affect patients' lives provides important information about the patients' health status and their perception of the treatment regime [22]. Moreover, this information helps enable clinicians to tailor treatment to the individual patient's needs.

Despite increasing interest in evaluating the impact of dyspepsia symptoms on patients' daily lives, measuring this impact in clinical trials can be problematic due to a paucity of validated instruments [23]. Furthermore, clinical trials are increasingly being conducted as multinational studies. This requires the availability of validated non-English language versions of the instrument to be used. Three patient-reported outcomes (PRO) instruments have been validated in patients with dyspepsia in clinical trials: the Severity of Dyspepsia Assessment (SODA) [24], the Nepean Dyspepsia Index (NDI) [25] and the Dyspepsia Symptom Severity Index (DSSI) [26]. However, all three instruments have only been validated as English-language versions. A further concern is that the NDI has not been validated for treatment effects, and the SODA was validated in a non-representative patient population comprising 96% men [24].

To be a viable measure of treatment outcome in clinical trials, PRO instruments need to be extensively documented to meet scientific standards [27,28] and to satisfy regulatory criteria, particularly with regard to claims for labelling and promotion [28,29]. The regulatory criterion is twofold: linguistic cross-cultural adaptation, and psychometric documentation. The US Food and Drug Administration (FDA) has recently issued a draft guidance on PRO measures [28]. In line with these guidelines, instruments need to show reliability, validity and an ability to detect change.

This report aims to document the psychometric characteristics of two PRO instruments, the Gastrointestinal Symptom Rating Scale (GSRS) and the Quality of Life in Reflux and Dyspepsia questionnaire (QOLRAD), in patients with dyspepsia. The GSRS and the QOLRAD were developed in US English. Several translations of both questionnaires have been psychometrically validated previously in patients with GERD [30-35]. In addition, English versions of the questionnaires have been used in dyspeptic populations (AstraZeneca, data on file). For the present report, a series of studies was undertaken in patients with dyspepsia in South Africa, Germany, Hungary, Italy, Poland and Spain. To enable comparisons between the various countries studied, results from all countries are presented here together in the current paper.

## Methods

### Patients

Consecutive patients with predominant symptoms of dyspepsia attending gastroenterology clinics between December 2000 and August 2003 were included in the study. In Germany and Spain, patients were also included from primary care. Dyspepsia was defined as persistent and/or recurrent pain or discomfort centred in the upper abdomen. Inclusion criteria required a history of episodes of dyspepsia for 6 months or longer, and episodes of dyspepsia on at least 1 day during the previous 7 days. The diagnosis based on the patient reports, and was verified at the discretion of the investigator. Where the diagnosis was uncertain, patients were not included. No physical examinations were performed. The following were exclusion criteria: GERD, irritable bowel syndrome, peptic ulcer disease, dementia or any other significant medical, psychiatric or surgical disease. Patients receiving daily treatment with acetyl salicylic acid or other nonsteroidal anti-inflammatory drugs were also excluded. Patients had to be able to complete the PRO instruments unaided, as no proxy assessment or interpreter was allowed. The administration of the questionnaires took place at visit 1 before any medical procedures were performed. Patients who were judged to be in stable phase were scheduled for a second visit. The second visit occurred 7 days after visit 1. The study was approved by local ethics committees in all countries requiring such approval for a non-drug related study. Good Clinical Practice was followed and the patients were free to discontinue participation in the study at any time.

### Demographic and clinical variables

Clinicians recorded patient demographics (including age, sex, ethnicity, and marital and employment status), medical history (including history of gastrointestinal diseases) and frequency of dyspepsia symptoms (number of days with episodes during the last 7 days). Investigators also assessed the severity of patients' dyspepsia symptoms by asking the patients and then recording the answer using a four-point graded scale (0 = none: no symptoms; 1 = mild: awareness of sign or symptom, but easily tolerated; 2 = moderate: discomfort sufficient to cause interference with normal activities; 3 = severe: incapacitating with inability to perform normal activities). All data were recorded in a case-report form.

### PRO instruments

Patients completed two disease-specific and two generic PRO instruments: the GSRS, the dyspepsia version of the QOLRAD, the 36-Item Short-Form Health Survey (SF-36), and the Hospital Anxiety and Depression scale (HAD) (except in South Africa, where patients were not given the HAD). Questionnaires were completed in an electronic data capture (EDC) device (Apple Newton Pad, Apple Computers Inc, Cupertino, CA, USA), except in South Africa, where paper and pencil were used. The method of using electronic patient-reported outcomes (ePRO) for recording of symptoms and health-related quality of life has previously been shown to improve the quality of the data and to be well received by patients [36]. Study personnel were trained in how to use the electronic device and how to instruct the patients in a standardized way to minimize bias and enhance compliance.

#### Gastrointestinal Symptom Rating Scale (GSRS)

The GSRS is a disease-specific instrument of 15 items combined into five symptom clusters depicting Reflux, Abdominal pain, Indigestion, Diarrhoea and Constipation. The GSRS has a seven-point graded Likert-type scale where 1 represents absence of troublesome symptoms and 7 represents very troublesome symptoms. The reliability and validity of the GSRS are well-documented [37], and norm values for a general population are available [38].

#### Quality of Life in Reflux and Dyspepsia questionnaire (QOLRAD)

The dyspepsia version of the QOLRAD is a disease-specific instrument, including 25 items combined into five dimensions: Emotional distress, Sleep disturbance, Vitality, Food/drink problems and Physical/social functioning. The questions are rated on a seven-point graded Likert scale; lower values indicate a more severe impact on daily functioning. The reliability and validity of the QOLRAD have been documented in patients with dyspepsia [39]. Previous studies have shown that a difference of approximately 0.5 points represents a clinically relevant change [40]. The factor structure of the QOLRAD has been replicated for several language versions [41].

#### 36-item Short-Form Health Survey (SF-36)

The SF-36 is an extensively used generic questionnaire containing 36 items clustered into eight dimensions (Bodily pain, General health, Mental health, Physical functioning, Role – emotional, Role – physical, Social functioning, Vitality). Item scores for each dimension are coded, summed and transformed to a scale from 0 (worst possible health status) to 100 (best possible health status). The SF-36 is well-documented in terms of reliability and validity in all available language versions [42,43]. This study used the acute version of the SF-36 covering a 1-week recall period [44].

#### Hospital Anxiety and Depression scale (HAD)

The HAD is a screening tool developed for use in medical settings. It consists of 14 items divided into two subscales for anxiety (seven items) and depression (seven items), in which the patient rates each item on a four-point scale. The higher scores indicate the presence of problems. A cut-off of > 11 implies definite cases of anxiety or depression, a cut-off of 8–10 a probable case, and < 7 not a case. The validity and reliability of the HAD have been reported in several studies [45,46]. The HAD was not used in South Africa because the Afrikaans translation was not available at the start of the study.

All instruments have been translated and linguistically validated according to international guidelines [47]. The linguistic validation of a questionnaire is not a literal translation of the original questionnaire, but the production of a translation, which is conceptually equivalent to the original and culturally acceptable in the country in which the translation will be used. This translation process includes forward- and back-translations by different, independent translators. Several translations of the GSRS and the QOLRAD are available, including Afrikaans, German, Hungarian, Italian, Polish and Spanish versions, and all have been psychometrically validated previously in patients with GERD [30-35].

### Psychometric evaluation of the GSRS and the QOLRAD

#### Reliability

*Internal consistency *refers to the extent to which the items within each dimension are interrelated. Cronbach's alpha is the most widely used measurement for assessing internal consistency reliability [48]. A high alpha coefficient (≥ 0.70) suggests that the items within a dimension measure the same construct and supports the construct validity.

*Test-retest reliability *measures the stability of a score derived from serial administrations of a measure by the same rater. Repeated measurements are made in the same individuals, at a time interval long enough to ensure independence. Here, patients whose symptoms were judged to be stable, and in whom the treatment – not study mandated – remained unchanged, were assessed between visits 1 and 2. An intraclass correlation coefficient (ICC) above 0.70 was considered to be acceptable [49].

#### Construct validity

Construct validity is concerned with whether the indicator actually measures the underlying attribute. The construct validity was examined by convergent, discriminant and known-groups validity.

*Convergent validity *consists of showing that a postulated dimension of the instrument correlates appreciably with all other dimensions that theory suggests should be related to it. Here, it was examined by: (a) correlating the GSRS with the QOLRAD; (b) correlating the GSRS and the QOLRAD with the SF-36 dimensions; and (c) correlating the GSRS and the QOLRAD with the HAD (except in South Africa) and with clinician-rated severity of dyspepsia symptoms. Similar dimensions in these instruments were expected to have high correlations with each other as shown by Pearson's product moment correlation. A strong correlation was considered to be over 0.60, a moderate between 0.30 and 0.60 and a low (very low) correlation below 0.30 [50]. Low correlations were expected between those dimensions that are theoretically unrelated constructs, thereby testing the *discriminant validity *of the instruments.

*Known-groups validity *consists of showing that an instrument can differentiate between groups of patients whose health status differs according to the characteristics of patients' disease, in this case clinician-rated frequency and severity of dyspepsia symptoms [51,52].

### Statistical methods

Statistical analyses were performed using Statistical Analysis System (SAS, version 8.02) [53]. Bonferroni correction was used to adjust for the multiplicity (significance level, P < 0.0003), corresponding to a correlation of 0.32 or above [54]. If data were missing from one or more item in a PRO instrument, the mean of the completed items in the same dimension was used provided that more than half of the items in that dimension had been completed [55]. The percentage of missing data in the GSRS and QOLRAD, respectively, were as follows: South Africa, 2.7% and 2.7%; Germany, 6.4% and 12.7%; Hungary, 8.8% and 8.8%; Italy, 6.3% and 1.1%; Poland, 0.7% and 0.7%; Spain, 0.6% and 0.6%. In cases where more than 6% of the data were missing (Germany, Hungary and Italy), characteristics (age, gender, duration and frequency of dyspepsia symptoms) of patients with missing data were compared with those who completed the questionnaires fully. There were no significant differences between the two groups.

## Results

### Demographic and clinical characteristics

A total of 853 patients with dyspepsia were included in the study. Patient demographics and clinical characteristics are shown in Table 1. Patients' ages ranged from 18 to 79 years (range, mean age: 42.2–48.3 years; standard deviation [SD]: 11.8–15.9 years), and the majority (61.1–72.1%) of patients were female. Virtually all participants were Caucasian, except in South Africa, where 89% of patients were of mixed ethnic origins. In all countries, more than half of patients were married (range: 59.5–69.7%). Full-time employment was highest in Hungary and lowest in South Africa and Spain (overall range: 41.4–64.0%).

**Table 1:** Patient demographics and clinical data for all countries (total N = 853). Values are in percentages unless otherwise specified.

**Variables**	**South Africa**	**Germany**	**Hungary**	**Italy**	**Poland**	**Spain**
Number of patients						
visit 1	111	141	136	175	135	155
visit 2	54	54	82	101	78	72
Mean age (years), (SD)	42.2 (11.8)	43.8 (14.9)	45.1 (14.2)	45.8 (15.4)	45.0 (14.0)	48.3 (15.9)
Female	68.5	68.8	72.1	61.1	66.7	67.7
Caucasian	11	98	100	100	100	100
Married	59.5	61.7	69.1	69.7	65.9	69.0
Full-time employed	41.4	61.0	64.0	48.6	52.6	43.9
Duration of current episode of dyspepsia symptoms > 1 month	71.2	53.2	72.8	58.3	65.1	72.3
Duration of disease > 5 years	26.1	41.8	24.3	19.4	40.0	49.7
Severity of symptoms last 7 days at least moderate	95.5	89.3	73.5	75.4	77.8	69.7
Symptoms on at least 5 days in previous week	64.9	61.0	47.1	28.6	62.2	48.4
No comorbidity	59.4	80.8	69.8	84.0	73.3	31.6
Concomitant medication						
No current medication	33.3	71.6	37.5	58.3	39.2	33.5
Proton pump inhibitors	21.6	15.6	25.7	6.8	27.4	20.6
Predominant dyspepsia symptom						
Abdominal pain	70.3	50.4	58.8	38.3	47.4	20.6
Abdominal discomfort	29.7	46.1	30.1	58.3	51.9	79.4
Previous peptic ulcer and/or ulcerative reflux esophagitis	14.4	4.3	6.6	9.1	8.1	5.2
Emotional problems, past 5 years	33.3	2.8	5.1	8.0	13.3	38.7

SD, standard deviation.

The majority (59.3–70.9%) of patients had moderate dyspepsia symptoms over the past week, except in South Africa, where the majority (55%) had severe symptoms. In terms of frequency, the majority (61.0–64.9%) of patients in South Africa, Germany and Poland had symptoms on at least five days in the previous week, as did approximately half of patients in Hungary (47.1%) and Spain (48.4%). In Italy, the majority (53.7%) of patients had symptoms on 3 to 4 days in the previous week.

### Psychometric evaluation

#### Reliability

Cronbach's alpha scores ranged from 0.43 (Abdominal pain, German version) to 0.87 (Constipation, Polish version) for the GSRS, and from 0.79 (Vitality, Afrikaans version) to 0.95 (Emotional distress, Spanish version) for the QOLRAD (Table 2). Scores were high (range: 0.72–0.87) for all language versions of the GSRS in the Indigestion, Diarrhoea and Constipation domains, thus demonstrating high internal consistency. Scores in the Reflux domain were also high for the German (0.72), Hungarian (0.73) and Italian (0.84) versions of the GSRS, but were below 0.7 for the Afrikaans, Polish and Spanish versions. Scores in the Abdominal pain domain were below 0.7 for all six countries. For the QOLRAD, Cronbach's alpha scores were high for all language versions in all domains.

**Table 2:** Cronbach's alpha at visit 1.

	**Translation**					
**Questionnaire and domain**	**Afrikaans**	**German**	**Hungarian**	**Italian**	**Polish**	**Spanish**

**GSRS**	(N = 108)	(N = 132)	(N = 124)	(N = 165)	(N = 134)	(N = 154)
Reflux	0.61	0.72	0.73	0.84	0.49	0.67
Abdominal pain	0.60	0.43	0.59	0.65	0.49	0.45
Indigestion	0.76	0.79	0.74	0.74	0.73	0.78
Diarrhoea	0.75	0.84	0.79	0.72	0.78	0.82
Constipation	0.82	0.81	0.79	0.76	0.87	0.75
						
**QOLRAD**	(N = 108)	(N = 123)	(N = 124)	(N = 174)	(N = 134)	(N = 154)
Emotional distress	0.91	0.94	0.94	0.93	0.92	0.95
Food/drink problems	0.83	0.90	0.90	0.88	0.86	0.91
Physical/social functioning	0.84	0.90	0.89	0.89	0.86	0.91
Sleep disturbance	0.88	0.89	0.94	0.89	0.91	0.93
Vitality	0.79	0.90	0.87	0.80	0.87	0.89

GSRS, Gastrointestinal Symptom Rating Scale; QOLRAD, Quality of Life in Reflux and Dyspepsia questionnaire.

Intraclass correlation coefficients for the two visits spaced about 1 week apart ranged from 0.36 (Abdominal pain, German version) to 0.75 (Indigestion, Hungarian and Spanish versions) for the GSRS, and from 0.41 (Vitality, Polish version) to 0.82 (Sleep disturbance, German version) for the QOLRAD (Table 3). Intraclass correlation coefficients were high (> 0.7) for the Hungarian, Polish and Spanish versions of the GSRS in the Indigestion domain, for the Hungarian version in the Diarrhoea domain, and for the Afrikaans, Hungarian, Polish and Spanish versions in the Constipation domain. Intraclass correlation coefficients for the GSRS Reflux and Abdominal pain domains were below 0.7 for all countries. For the QOLRAD, intraclass correlation coefficients were high for all domains in the Hungarian version, and for all domains except Food/drink problems in the German version. Intraclass correlation coefficients were below 0.7 for all QOLRAD domains in the Afrikaans and Polish versions, and for all but one (Food/drink problems) domain in the Spanish version. Test-retest reliability was not assessed in Italy, as a follow-up with the physicians revealed that many patients were not in a stable phase between visits 1 and 2: some patients changed their self-medication between the two visits, and some were endoscoped.

**Table 3:** Test-retest reliability (intraclass correlation coefficient [ICC]) for Gastrointestinal Symptom Rating Scale (GSRS) and Quality of Life in Reflux and Dyspepsia questionnaire (QOLRAD) domains between visits 1 and 2.

**Questionnaire and domain**	**Translation**					
	**Afrikaans**	**German**	**Hungarian**	Italian*	**Polish**	**Spanish**

**GSRS**	(N = 108)	(N = 132)	(N = 124)	(N = 165)	(N = 134)	(N = 154)
Reflux	0.37	0.66	0.59	N/A	0.61	0.48
Abdominal pain	0.55	0.36	0.57	N/A	0.45	0.62
Indigestion	0.46	0.61	0.75	N/A	0.71	0.75
Diarrhoea	0.56	0.67	0.70	N/A	0.63	0.38
Constipation	0.71	0.60	0.72	N/A	0.72	0.72
						
**QOLRAD**	(N = 108)	(N = 123)	(N = 124)	(N = 174)	(N = 134)	(N = 154)
Emotional distress	0.51	0.75	0.70	N/A	0.56	0.65
Food/drink problems	0.51	0.62	0.79	N/A	0.62	0.75
Physical/social functioning	0.53	0.73	0.74	N/A	0.68	0.69
Sleep disturbance	0.62	0.73	0.82	N/A	0.61	0.66
Vitality	0.46	0.71	0.74	N/A	0.41	0.66

*In the Italian translation, test-retest reliability was not analyzed because the patients were not in a stable phase between visit 1 and 2.

#### Construct validity

##### Correlation of the GSRS with the QOLRAD

In the Afrikaans versions, correlations (as defined by a Pearson's product moment correlation = 0.3) were only observed between the GSRS Abdominal pain domain and the QOLRAD Emotional distress and Physical/social functioning domains. In the German versions, the GSRS Abdominal pain and Indigestion domains correlated with all QOLRAD domains, but the GSRS Reflux domain correlated only with the QOLRAD Food/drink problems domain. The relevant GSRS domains Reflux, Abdominal pain and Indigestion correlated with all QOLRAD domains in the Hungarian version of the questionnaires. This was also the case for the Italian versions of the questionnaires, with the exception of the GSRS Reflux and Indigestion domains, which did not correlate with the QOLRAD Food/drink problems domain. In the Polish versions of the questionnaires, the GSRS Reflux and Abdominal pain domains correlated with the QOLRAD Food/drink problems, Physical/social functioning, and Sleep disturbance domains. As was the case with the Italian versions, the GSRS Reflux, Abdominal pain and Indigestion domains correlated with all QOLRAD domains in the Spanish version of the questionnaires.

##### Correlation of the GSRS with the SF-36

Each domain of the GSRS correlated negatively with each domain of the SF-36 in all language versions of the questionnaires. Significant correlations between GSRS domains and SF-36 domains (Pearson's product moment correlation = 0.3) are shown in Table 4.

**Table 4:** Significant correlations between Gastrointestinal Symptom Rating Scale (GSRS), Quality of Life in Reflux and Dyspepsia Questionnaire (QOLRAD) and 36-item Short-Form Health Survey (SF-36) domains (Pearson's product moment correlation ≥ 3).

**Questionnaire and domain**	**SF-36 domain and correlation coefficient**					
	Afrikaans*	**German**	**Hungarian**	**Italian**	**Polish**	**Spanish**

**GSRS**						
Reflux	--	GH (0.32), PF (0.30)	BP (0.35), GH (0.41), MH (0.38), PF (0.46), RP (0.30), SF (0.34), V (0.39)	--	--	BP (0.35)
Abdominal pain	BP (0.45), PF (0.33)	BP (0.40), MH (0.45), PF (0.34), RE (0.36), RP (0.43), SF (0.31), V (0.40)	BP (0.54), GH (0.32), MH (0.45), PF (0.43), RE (0.30), RP (0.46), SF (0.50), V (0.52)	BP (0.43), MH (0.36), RE (0.36), SF (0.37)	BP (0.36), RP (0.35)	BP (0.37), MH (0.40), RE (0.34), SF (0.35), V (0.34)
Indigestion	BP (0.39), PF (0.34)	GH (0.35), RE (0.31), V (0.30)	BP (0.53), GH (0.35), MH (0.42), PF (0.43), RE (0.30), RP (0.42), SF (0.47), V (0.44)	MH (0.30), RE (0.31), SF (0.33),	--	BP (0.39), GH (0.32), MH (0.32), V (0.31)
Diarrhoea	--	GH (0.37), MH (0.35), PF (0.31), RE (0.30), SF (0.43)	GH (0.31), SF (0.37)	PF (0.30)	--	GH (0.31), MH (0.37), SF (0.30)
Constipation	BP (0.34)	SF (0.35)	BP (0.40), GH (0.41), MH (0.34), PF (0.46), RP (0.38), SF (0.40), V (0.33)	PF (0.39)	--	BP (0.30)
**QOLRAD**						
Emotional distress	BP (0.50), MH (0.35), RE (0.35), RP (0.43), SF (0.36), V (0.39)	BP (0.41), GH (0.40), MH (0.68), PF (0.48), RE (0.42), RP (0.60), SF (0.61), V (0.60)	BP (0.68), GH (0.59), MH (0.65), PF (0.52), RE (0.47), RP (0.55), SF (0.58), V (0.68)	BP (0.45), GH (0.49), MH (0.59), PF (0.36), RE (0.49), RP (0.47), SF (0.56), V (0.56)	BP (0.42), GH (0.36), MH (0.47), PF (0.34), RE (0.30), RP (0.37), SF (0.49), V (0.44)	BP (0.47), GH (0.43), MH (0.55), PF (0.33), RE (0.39), RP (0.42), SF (0.51), V (0.44)
Sleep disturbance	BP (0.39), GH (0.31), MH (0.35), RE (0.31), RP (0.41), SF (0.38), V (0.49)	BP (0.43), GH (0.30), MH (0.44), PF (0.48), RE (0.42), RP (0.60), SF (0.61), V (0.60)	BP (0.63), GH (0.56), MH (0.51), PF (0.64), RE (0.51), RP (0.64), SF (0.49), V (0.59)	BP (0.46), GH (0.48), MH (0.46), PF (0.37), RE (0.47), RP (0.43), SF (0.51), V (0.50)	BP (0.43), MH (0.33), PF (0.32), RE (0.30), RP (0.41), SF (0.38), V (0.36)	BP (0.39), GH (0.34), MH (0.50), PF (0.37), RE (0.33), RP (0.44), SF (0.44), V (0.47)
Food/drink problems	BP (0.32), GH (0.31), V (0.41)	BP (0.46), GH (0.31), MH (0.50), PF (0.50), RE (0.42), RP (0.60), SF (0.45), V (0.48)	BP (0.62), GH (0.40), MH (0.44), PF (0.49), RE (0.43), RP (0.58), SF (0.49), V (0.49)	BP (0.59), GH (0.50), MH (0.46), RE (0.45), RP (0.46), SF (0.52), V (0.51)	BP (0.40), GH (0.31), MH (0.37), PF (0.33), RE (0.33), RP (0.40), SF (0.41), V (0.32)	BP (0.38), MH (0.37), RE (0.37), RP (0.35), SF (0.39), V (0.33)
Physical/social functioning	BP (0.44), MH (0.39), PF (0.32), RE (0.44), RP (0.47), SF (0.48), V (0.39)	BP (0.49), GH (0.38), MH (0.56), PF (0.62), RE (0.43), RP (0.69), SF (0.64), V (0.52)	BP (0.76), GH (0.52), MH (0.61), PF (0.66), RE (0.54), RP (0.73), SF (0.69), V (0.68)	BP (0.58), GH (0.49), MH (0.51), PF (0.45), RE (0.56), RP (0.60), SF (0.69), V (0.56)	BP (0.45), GH (0.46), MH (0.50), PF (0.46), RE (0.40), RP (0.53), SF (0.55), V (0.42)	BP (0.46), GH (0.45), MH (0.48), PF (0.49), RE (0.35), RP (0.55), SF (0.61), V (0.46)
Vitality	BP (0.46), MH (0.32), RE (0.31), RP (0.35), SF (0.34), V (0.36)	BP (0.50), GH (0.31), MH (0.59), PF (0.46), RE (0.47), RP (0.66), SF (0.49), V (0.63)	BP (0.72), GH (0.55), MH (0.63), PF (0.60), RE (0.51), RP (0.65), SF (0.60), V (0.71)	BP (0.54), GH (0.47), MH (0.50), PF (0.33), RE (0.52), RP (0.49), SF (0.53), V (0.59)	BP (0.41), GH (0.35), MH (0.44), PF (0.27), RE (0.26), RP (0.40), SF (0.50), V (0.44)	BP (0.43), GH (0.39), MH (0.52), PF (0.36), RE (0.38), RP (0.44), SF (0.51)

BP, Bodily pain; GH, General Health; MH, Mental Health; PF, Physical functioning; RE, Role – Emotional; RP, Role – Physical; SF, Social Functioning; V, Vitality

##### Correlation of the QOLRAD with the SF-36

QOLRAD domains correlated significantly with the majority of SF-36 domains in most language versions. Significant correlations between QOLRAD domains and SF-36 domains (Pearson's product moment correlation = 0.3) are shown in Table 4.

##### Correlation of the GSRS with the HAD

Correlations between GSRS domains and the HAD anxiety and depression scores are shown in Table 5. The GSRS Abdominal pain and Indigestion domains correlated significantly with the HAD anxiety score in the German, Spanish and Hungarian populations. The GSRS Reflux domain correlated with the HAD depression score only in the Hungarian population. (Note: HAD was not assessed in South Africa.)

**Table 5:** Pearson's product moment correlations between Gastrointestinal Symptom Rating Scale (GSRS) and Quality of Life in Reflux and Dyspepsia Questionnaire (QOLRAD) domains and Hospital Anxiety and Depression Scale (HAD).

**Questionnaire and domain**	**Translation**					
	Afrikaans*	**German**	**Hungarian**	**Italian**	**Polish**	**Spanish**

**GSRS**	(N = 108)	(N = 132)	(N = 124)	(N = 165)	(N = 134)	(N = 154)
Reflux	N/A	A: 0.29,	A: **0.44**,	A: 0.16	A: 0.22	A: 0.15
		D: 0.14	D: **0.44**	D: 0.01	D: 0.04	D: 0.17
Abdominal pain	N/A	A: **0.48**,	A: **0.38**,	A: 0.29	A: 0.15	A: **0.35**
		D: 0.18	D: **0.38**	D: 0.18	D: 0.03	D: 0.28
Indigestion	N/A	A: **0.44**,	A: **0.38**,	A: 0.27	A: 0.09	A: **0.33**
		D: 0.14	D: **0.36**	D: 0.15	D: 0.13	D: 0.25
Diarrhoea	N/A	A: **0.35**,	A: 0.26,	A: 0.10	A: 0.24	A: **0.31**
		D: 0.21	D: 0.19	D: -0.01	D: 0.21	D: 0.26
Constipation	N/A	A: **0.32**,	A: **0.39**,	A: 0.25	A: -0.00	A: 0.25
		D: 0.07	D: 0.48	D: 0.11	D: -0.01	D: 0.16
						
**QOLRAD**	(N = 108)	(N = 123)	(N = 124)	(N = 174)	(N = 134)	(N = 154)
Emotional distress	N/A	A: **0.49**,	A: **0.67**,	A: **0.67**	A: **0.40**	A: **0.41**
		D: **0.36**	D: **0.57**	D: **0.49**	D: **0.34**	D: **0.44**
Food/drink problems	N/A	A: **0.35**,	A: **0.48**,	A: **0.51**	A: **0.35**	A: **0.30**
		D: 0.18	D: **0.47**	D: **0.47**	D: 0.27	D: 0.28
Physical/social functioning	N/A	A: **0.36**,	A: **0.60**,	A: **0.64**	A: **0.43**	A: **0.37**
		D: 0.28	D: **0.64**	D: **0.53**	D: **0.38**	D: **0.48**
Sleep disturbance	N/A	A: 0.29,	A: **0.59**,	A: **0.53**	A: 0.29	A: **0.39**
		D: 0.14	D: **0.59**	D: **0.41**	D: 0.27	D: **0.36**
Vitality	N/A	A: **0.41**,	A: **0.64**,	A: **0.59**	A: 0.30	A: **0.43**
		D: **0.32**	D: **0.61**	D: **0.46**	D: 0.30	D: **0.42**

*The HAD was not used in South Africa. A = HAD anxiety score; D = HAD depression score. Strong correlation, > 0.60; moderate correlation, 0.30–0.60; low (very low) correlation, < 0.30.

##### Correlation of the QOLRAD with the HAD

Correlations between QOLRAD domains and the HAD anxiety and depression scores are shown in Table 5. All QOLRAD domains correlated with the HAD anxiety scores in the Hungarian, Italian and Spanish versions of the questionnaire, and all except Sleep disturbance correlated with the HAD anxiety scores in the German and Polish versions. Furthermore, all QOLRAD domains correlated with the HAD depression scores in the Italian and Hungarian versions of the questionnaire, and all except Food/drink problems correlated with the HAD depression score in the Spanish version.

##### Correlation of the GSRS with physician-assessed severity of symptoms

The relevant GSRS Abdominal pain domain correlated significantly with physician-assessed severity of symptoms in the Afrikaans version of the GSRS. The GSRS Abdominal pain and Indigestion domains correlated significantly with physician-assessed severity of symptoms in the Spanish version of the questionnaire, and with physician-assessed frequency and severity in the Hungarian version. Taken together, these correlations show convergent validity.

##### Correlation of the QOLRAD with physician-assessed severity of symptoms

All QOLRAD domains correlated with physician-assessed severity and frequency of symptoms in the Hungarian version of the questionnaire, and all domains except Sleep disturbance correlated with physician-assessed severity and frequency in the Spanish version. In the German versions, the QOLRAD emotional distress domain correlated with physician-assessed frequency of symptoms, and the QOLRAD Sleep disturbance domain correlated with physician-assessed severity. The QOLRAD Vitality domain correlated with physician-assessed severity in the Italian version of the questionnaire. In the Polish version, the QOLRAD Physical/social functioning and Vitality domains correlated with physician-assessed frequency and severity of symptoms, and the QOLRAD Emotional distress domain correlated with physician-assessed frequency of symptoms.

##### Known-groups validity of the GSRS and the QOLRAD

All domains of the GSRS and the QOLRAD questionnaires were able to differentiate between groups of patients whose health status differed according to the physician-assessed overall frequency and severity of dyspepsia symptoms, thereby confirming the known-groups validity of the instruments. GSRS scores increased with increasing frequency and severity of dyspepsia symptoms, while QOLRAD scores decreased with increasing frequency and severity of symptoms (representing a decrease in daily functioning) (results not shown).

## Discussion

The GSRS and the QOLRAD are two of the most established, validated, reliable and responsive disease-specific instruments available for assessing gastrointestinal symptoms and their impact on patients' daily functioning [37,39]. Both questionnaires have been proven to have very good psychometric characteristics when tested in clinical trials in patients with GERD and dyspepsia [20,56,57]. This paper documents the psychometric validation of the Afrikaans, German, Hungarian, Italian, Polish and Spanish translations of the GSRS and the QOLRAD in patients with dyspepsia. The study was conducted mainly in gastroenterology centres and, therefore, results are particular to patients referred for gastroenterological investigation. Because of the standardized methodology used, results from all countries are directly comparable.

Questionnaires were completed in an electronic data capture device (ECD), which ensures that all questions are answered in full. Although ECD technology is becoming an increasing part of clinical trials, it is not yet widely available, and certainly not that widespread in clinical practice. Thus, in South Africa, paper versions of the questionnaires were used for the present study, and this may have affected the results obtained in this country, especially as the use of ECD technology has been shown to increase patient compliance and improve the quality of the data [36].

The demographic and clinical characteristics of the patient populations were comparable in the different countries studied in terms of the number of patients recruited, their age, gender, symptom status and medication use. The Afrikaans patient population had the highest incidence of abdominal pain as the predominant dyspeptic symptom as well as the highest proportion of patients with a history of previous peptic ulcer and/or ulcerative reflux esophagitis. This was probably due to the fact that all South African subjects were recruited from a single gastroenterology clinic in a healthcare system that may refer only the most severely affected patients.

In terms of psychometric characteristics, internal consistency was high in all domains of all language versions of the QOLRAD, thus supporting construct validity. Internal consistency was also high in the Indigestion, Diarrhoea and Constipation domains of the GSRS in all language versions. However, although abdominal pain is the typical dyspepsia symptom, internal consistency was only moderate in the Abdominal pain domain of the GSRS. This generally low internal consistency in the Abdominal pain domain probably reflects the difficulty of measuring this often fluctuating symptom, as well as the fact that the GSRS Abdominal pain domain contains only two items. In previous international studies conducted in patients with GERD, internal consistency also tended to be lower in the Abdominal pain domain of the GSRS than in its other domains [30-35].

Test-retest reliability was good for most or all domains in the Hungarian and German translations of the QOLRAD, but only for one domain in the Spanish version and for no domains in the Afrikaans and Polish versions of the QOLRAD. It is not clear why the test-retest reliability was low in these patient populations, although it may be an indication that the frequency, severity and impact of abdominal symptoms were not as stable on a week-to-week basis in these patients as was estimated by the investigators. No test-retest was performed in Italy, so it was not possible to assess test-retest reliability for the Italian version of the QOLRAD. Previous international studies in patients with GERD have yielded acceptable test-retest reliability for the German (ICC: 0.70–0.84), Spanish (ICC: 0.79–0.85) and Afrikaans (ICC: 0.71–0.82) versions of the QOLRAD, but only for some domains of the Polish version (ICC: 0.51–74) of the QOLRAD [30-35]. In the GSRS, test-retest reliability was acceptable in the Indigestion and Constipation domains in most language versions. However, reliability was lower in the Abdominal pain domains in all countries assessed. As with internal consistency, the generally low test-retest reliability in the Abdominal pain domain is most likely due to the GSRS Abdominal pain domain containing only two items, as well as the complexity of measuring this variable symptom. It is also likely that at least some of the patients deemed stable by the physicians experienced small changes in their symptoms that were picked up by the PROs.

In terms of construct validity, all GSRS domains correlated with all QOLRAD domains. Correlation was significant for the relevant GSRS Abdominal pain domain and most or all QOLRAD domains in the majority of the different language versions. The GSRS Abdominal pain domain also correlated significantly with the relevant SF-36 Bodily pain domain in all language versions. All QOLRAD domains correlated significantly with the majority of SF-36 domains in most language versions. Both the GSRS and the QOLRAD were able to differentiate between patients whose health status differed with regard to frequency and severity of symptoms, thereby confirming the known-groups validity of the instruments. Known-groups validity has also been confirmed for several translations of the GSRS and the QOLRAD in patients with GERD [30-32,35]. Confirmatory factor analysis has been shown to support the validity of the Scandinavian language versions of the GSRS and the QOLRAD in patients with GERD [41]. Future studies could use this approach to further assess the validity of the additional language versions presented here.

The relevant GSRS Abdominal pain domain correlated with the HAD anxiety score in most of the language versions assessed, and most of the QOLRAD domains correlated with the HAD anxiety score in all language versions assessed. Dyspepsia tends to be associated with anxiety and depression [58]. Previous reports are somewhat contradictory on the role of psychological morbidity in dyspepsia symptoms and healthcare-seeking behaviour [1,58], suggesting that additional research is required on this potentially important aspect of the disorder.

When comparing the frequency and severity of patient-reported symptoms with those assessed by the physician, correlations were only low to moderate. These discrepancies in patient-reported and observer-reported symptom status have been well-documented [59-61]. Our view is that the physician examination needs to be balanced with the patient-reported symptom status when deciding on patient management and outcomes [39,62].

In summary, all language versions of the QOLRAD showed good internal consistency and reliability, although only the German and Hungarian translations were able to demonstrate acceptable test-retest reliability. In general, all language versions of the GSRS were reliable and showed good internal consistency for the Indigestion, Diarrhoea and Constipation domains, but not for the Abdominal pain domain. Overall, the test-retest reliability of the GSRS was not acceptable.

## Conclusion

In conclusion, the German, Hungarian, Italian, Polish, Spanish and Afrikaans versions of GSRS and QOLRAD are reliable and show good internal consistency for use in clinical trials. However, the test-retest reliability was low in most language versions. By evaluating symptoms and their impact on patients' lives, both these instruments help physicians interpret clinical benefits as outcomes of significance to patients. We believe that these results will further facilitate the use of these instruments in the clinical setting.

## Abbreviations

DSSI, Dyspepsia Symptom Severity Index

EDC, electronic data capture

ePRO, electronic patient-reported outcomes

FDA, Food and Drug Administration

GERD, gastroesophageal reflux disease

GSRS, Gastrointestinal Symptom Rating Scale

HAD, Hospital Anxiety and Depression scale

ICC, intraclass correlation coefficient

NDI, Nepean Dypepsia Index

PRO, patient-reported outcomes

QOLRAD, Quality of Life in Reflux and Dyspepsia questionnaire

SD, standard deviation

SF-36, 36-item Short-Form Health Survey

SODA, Severity of Dyspepsia Assessment

## Competing interests

All authors participated in the present study supported by AstraZeneca R&D, Mölndal, Sweden. Károly Kulich, Jonas Carlsson and Katarina Halling are employees of AstraZeneca R&D, Mölndal, Sweden. Ingela Wiklund was an employee of AstraZeneca R&D, Mölndal, Sweden at the time of the study. Ahmed Madisch, Franco Pacini, Jose Piqué, Jaroslaw Regula, Christoffel Johannes van Rensburg and László Újszászy have no additional financial or other relationships to disclose.

## Authors' contributions

All authors contributed to the study design, data analyses and interpretation of results. Ahmed Madisch, Franco Pacini, Jose Piqué, Jaroslaw Regula, Christoffel Johannes van Rensburg and László Újszászy coordinated the patient recruitment and data collection. Jonas Carlson provided statistical support. All authors provided comments and revisions on a first version of the manuscript drafted by Károly Kulich, Katarina Halling and Ingela Wiklund. All authors have seen and approved the final version of the manuscript.
